# Metal Nano/Microparticles for Bioapplications

**DOI:** 10.3390/ijms22094543

**Published:** 2021-04-27

**Authors:** Xuan-Hung Pham, Seung-min Park, Bong-Hyun Jun

**Affiliations:** 1 Department of Bioscience and Biotechnology, Konkuk University, Seoul 143-701, Korea; phamricky@gmail.com; 2 Department of Urology, Stanford University School of Medicine, Stanford, CA 94305, USA; sp293@stanford.edu; 3 Department of Radiology, Stanford University School of Medicine, Stanford, CA 94305, USA; 4 Molecular Imaging Program at Stanford, Stanford University School of Medicine, Stanford, CA 94305, USA

Nano/micro particles are considered to be the most valuable and important functional materials in the field of materials science and engineering [1,2]. As a field, metal nanoparticles (NPs) have been one of the most actively studied fields in nano/microtechnology, along with carbon materials, magnetic materials, and quantum dots [3,4]. The ability of metal nanoparticles to interact effectively with electromagnetic radiation makes them suitable for many biomedical applications, including molecular recognition, diagnosis, treatment, and evaluation of disease. The interaction with electromagnetic radiation causes a unique optical phenomenon, called localized surface plasmon resonance (LSPR), usually in the ultra-violet (UV), visible, and near-infrared (NIR) spectrum range. The LSPR frequency can be finely tuned based on various physicochemical conditions, such as the distance between nanoparticles and particle size and shape [5].

This Special Issue provides a range of original contributions detailing the synthesis, modification, properties, and applications of metal materials, particularly in nanomedicine. Eleven outstanding papers describing examples of the most recent advances in metal nano/microparticles are included.

Gold nanoparticles and silver nanoparticles have been widely employed for various biomedical applications, because of their superior plasmonic properties [1,6,7]. Their size and shape can be controlled easily during fabrication for the fine tuning of surface plasmon resonances [8,9]. The surface chemistry and modification of Au and Ag NPs have been well studied, enabling them to be adopted as biosensors based on their SPR band changes. Seong S. et al. reported densely immobilized gold-assembled silica nanostructures using the Au seed-mediated growth. The catalytic activity of SiO_2_@Au was twice better than that of HF-treated SiO_2_@Au. When SiO_2_@Au nanostructure was used as a surface enhanced Raman scattering (SERS) substrate, the signal of 4-aminophenol on the surface of SiO_2_@Au was also stronger than that of HF-treated SiO_2_@Au [10]. Kang et al. presented a template-assisted method to synthesize nanogap shell structures for biomolecular detections based on SERS. The interior nanogap containing a silver shell structure was fabricated on Ag NPs-coated silica, by adsorbing small aromatic thiol molecules on the Ag NPs. The NPs showed a high SERS enhancement factor and good signal uniformity. In a model study, a prostate-specific antigen was calibrated with a limit of detection of 2 pg/mL [11]. Huynh et al. reviewed metallic alloy nanoparticles that are synthesized by combining two or more metals. Bimetallic or trimetallic nanoparticles are considered more effective in various aspects than monometallic nanoparticles. The authors outlined the structure, synthesis method, properties, and biological applications of metallic alloy nanoparticles based on their plasmonic, catalytic, and magnetic characteristics [9].

Humans and the environment are becoming increasingly exposed to nanomaterials, raising concerns about their safety. Various nanomaterials have been investigated for antiviral and antibacterial effects. Mizielińska et al. reported the potential antibacterial activity of the coatings based on nanoparticles of ZnO, carvacrol, and geraniol [12]. Kong et al. reported a unique toxicity based on Co-NPs based on their sizes. Two different sizes of cobalt oxide nanoparticles were evaluated in the contexts of the activities of bacterial bioluminescence, enzyme function and biosynthesis of β-galactosidase, bacterial gene mutations, algal growth, and plant seed germination and root/shoot growth [13]. Han et al. reported the stress response of mouse embryonic fibroblasts exposed to polystyrene (PS) nanoplastics. They found that PS nanoplastics in the cytoplasm affect cellular functions, but mouse embryonic fibroblasts (MEFs) can overcome the stress caused by PS nanoplastic exposure [14].

Several innovative metal-based nanomaterials have been introduced in bioapplications. Anticancer properties of platinum nanoparticles and retinoic acid as a combination therapy for the treatment of human neuroblastoma cancer was reported by Gurunathan et al. The anticancer effects of platinum nanoparticles (PtNPs) and retinoic acid on neuroblastoma were assessed and they demonstrated that treatment of SH-SY5Y cells with the combination of PtNPs and RA resulted in improved anticancer effects [15]. Ag NPs have a potential to be used as a sunscreen ingredient. Ho et al. reported that Ag NPs effectively protects against UVB-induced skin damage both in cell cultures and mouse models. They claimed that AgNPs are feasible and safe as sunscreen ingredients for protection against UVB-induced skin damage [16].

Several novel metal related review papers were also included. Miguel et al. overviewed the current knowledge regarding the use of superparamagnetic iron oxide nanoparticles and essential oils on the prevention of microbial adherence and consequent biofilm formation with a goal of being applied on the surface of medical devices [17].

Innovative green synthesis of metallic nanoparticles was reviewed. Since recently conifer extracts have been found to be effective in synthesizing metallic nanoparticles, Bhardwaj et al. highlight the importance of conifers and its extracts in synthesis of metallic nanoparticles as well as unique applications [18]. Kumar et al. reviewed the potential activities of phytochemicals, and intend to summarize the different metallic nanoparticles synthesized using fruit extracts and their associated pharmacological activities such as anti-cancerous, antimicrobial, antioxidant, and catalytic efficiency [19].

This Special Issue highlights outstanding advances in the development of metal nano/microparticles for bioapplications. We hope that the Special Issue will be of great help to next-generation bioapplications.

## Data Availability

Not applicable.
